# Mitochondria mediates caspase-dependent and independent retinal cell death in *Staphylococcus aureus* endophthalmitis

**DOI:** 10.1038/cddiscovery.2016.34

**Published:** 2016-05-30

**Authors:** P K Singh, A Kumar

**Affiliations:** 1 Kresge Eye Institute/Department of Ophthalmology, Wayne State University, Detroit, MI, USA; 2 Department of Anatomy and Cell Biology, Wayne State University, Detroit, MI, USA; 3 Department of Immunology and Microbiology, Wayne State University, Detroit, MI, USA

## Abstract

Bacterial endophthalmitis, a vision-threatening complication of ocular surgery or trauma, is characterized by increased intraocular inflammation and retinal tissue damage. Although significant vision loss in endophthalmitis has been linked to retinal cell death, the underlying mechanisms of cell death remain elusive. In this study, using a mouse model of *Staphylococcus aureus* endophthalmitis and cultured human retinal Müller glia (MIO-M1 cell line), we demonstrate that *S. aureus* caused significant apoptotic cell death in the mouse retina and Müller glia, as evidenced by increased number of terminal dUTP nick end labeling and Annexin V and propidium iodide-positive cells. Immunohistochemistry and western blot studies revealed the reduction in mitochondrial membrane potential (JC-1 staining), release of cytochrome *c* into the cytosol, translocation of Bax to the mitochondria and the activation of caspase-9 and -3 in *S. aureus-*infected retina/retinal cells. In addition, the activation of PARP-1 and the release of apoptosis inducing factor from mitochondria was also observed in *S. aureus*-infected retinal cells. Inhibition studies using pan-caspase (Q-VD-OPH) and PARP-1 (DPQ) inhibitors showed significant reduction in *S. aureus*-induced retinal cell death both *in vivo* and *in vitro*. Together, our findings demonstrate that in bacterial endophthalmitis, retinal cells undergo apoptosis in the both caspase-dependent and independent manners, and mitochondria have a central role in this process. Hence, targeting the identified signaling pathways may provide the rationale to design therapeutic interventions to prevent bystander retinal tissue damage in bacterial endophthalmitis.

## Introduction

Apoptosis is a process of programmed cell death, which involves a sequence of events, including shrinkage of cytoplasm, condensation of nuclear chromatin with DNA fragmentation and segmentation of the cell into apoptotic bodies.^1,2^ This neat packaging of cellular components allows for the precise removal of tissue during developmental remodeling in the retina. Beyond this involvement in ocular development, apoptosis rarely occurs in a normal, healthy retina but has been implicated in both inherited and acquired retinal degenerations and various pathological conditions.^3^ The molecular pathogenesis of these retinal degenerations is still unclear, but apoptotic cell death remains the final outcome in many retinal diseases, ranging from glaucoma to age-related macular degeneration, retinitis pigmentosa and retinal detachment.^4–6^ Therefore, targeting retinal cell death using various neuro-protective therapies has been the subject of extensive research for the past decades, and yet the mechanisms underlying cell death in various retinal diseases, including glaucoma remains elusive.^7,8^ Similarly, very few studies have investigated retinal cell death under infectious conditions, such as bacterial endophthalmitis.^9,10^ Moreover, to our knowledge, none of the studies elucidated the detailed mechanisms of apoptotic retinal cell death in endophthalmitis.^11,12^ As our recent studies show increased terminal dUTP nick end labeling (TUNEL)-positive cells in the retina of *Staphylococcus aureus*^13^ and *Acinetobacter baumannii*^14^ infected mouse eyes, we sought to elucidate the fundamental mechanisms and potential check points in retinal cell death in bacterial endophthalmitis.

Apoptotic response can be evoked by a wide variety of stimuli such as death receptors, oxidative stress or microbial infection, and it involves several cellular molecules, including caspases, Bcl-2-like proteins, mitochondrial factors, and stress-activated protein kinases, and so on. Regardless of extrinsic or intrinsic pathways; the caspases have been shown to be key regulators of almost any form of apoptosis.^15^ The caspases, being the proteases, cleave multiple intracellular proteins resulting in the propagation and final execution of the apoptotic signals. Among the cellular organelles, the mitochondria are the central regulators of cell death during development and under pathological conditions.^16,17^ The mitochondria mediates apoptosis through the release of various pro-apoptotic factors, the best characterized of which is cytochrome *c*.^16,18,19^ The release of cytochrome *c* into the cytosol induces the formation of the apoptosome and activation of caspase-9 followed by activation of executioner caspase-3. However, several other apoptotic proteins capable of inducing cellular apoptosis in a caspase-independent manner also reside within mitochondria. These include apoptosis inducing factor (AIF), Omi/HtrA2 and endonuclease G.^20–24^ The release of these pro-death factors requires mitochondrial membrane permeabilization which is regulated by the Bcl-2 family. One of the pro-apoptotic Bcl-2 family proteins Bax has been shown to mediate these pathways.^25^ However, whether mitochondrial pathways have a role in retinal cell apoptosis in bacterial endophthalmitis is not known.

Our previous studies revealed an essential role of retinal residential cells (microglia and Müller glia) in providing retinal innate defense in endophthalmitis.^26–29^ The retinal glial cells, specifically the Müller glia were found to exhibit multiple mechanisms to kill the invading pathogen including the generation of reactive oxygen species (ROS) in response to *S. aureus* challenge.^29^ Although, the production of ROS by Müller glia may contribute towards direct antimicrobial effects, the excessive ROS levels are known to induce cell death.^30^ As the mitochondria are the major source of ROS generation, we hypothesized that mitochondria have a role in the induction of retinal cell apoptosis in bacterial endophthalmitis. Here, we elucidated the molecular mechanisms of retinal cell death using both *in vivo* and *in vitro* models of *S. aureus* endophthalmitis. Our data provide the evidence for induction of apoptosis by mitochondrial-mediated caspase-dependent and -independent pathways. Furthermore, targeting of both pathways attenuated apoptosis in *S. aureus*-infected mouse retina, suggesting the therapeutic potential of these approaches in ameliorating retinal tissue damage.

## Results

### *S. aureus* induces apoptotic cell death in the mouse retina and retinal Müller glia

**Figure 1:**
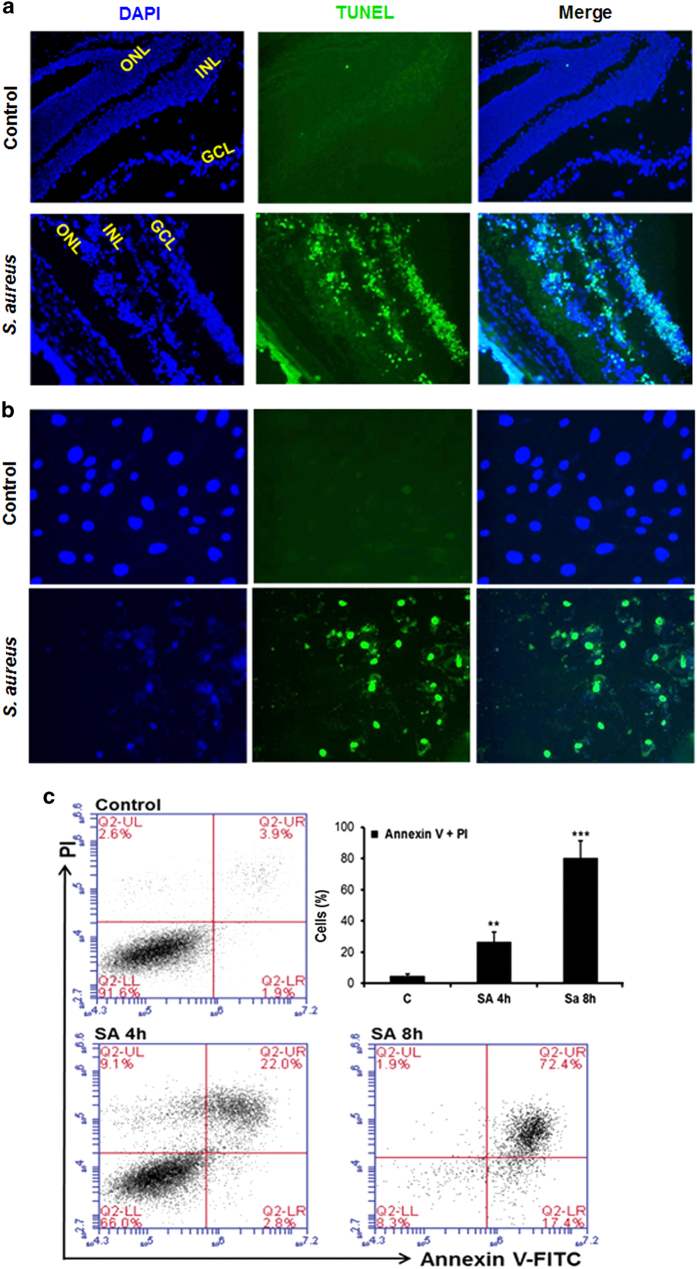
*S. aureus* induces apoptosis in the mouse retina and retinal Müller glia. The right eyes of C57BL/6 mice (*n*=4) were injected intravitreally with 5000 c.f.u. of *S. aureus* (SA) RN6390, and the left eyes were injected with PBS (control). After 24 h, eyes were enucleated and embedded in OCT, and retinal cryosections were subjected to TUNEL staining, (blue, DAPI nuclear stain; green, TUNEL-positive cells) (**a**). In an *in vitro* experiment, human retinal Müller glia (MIO-M1 cell line) were left uninfected (control) or challenged with *S. aureus* for 8 h at the multiplicity of infection of 10 : 1. The control and *S. aureus*-infected cells were fixed, permeabilized and subjected to TUNEL staining (**b**). For quantitative analysis of apoptotic cells, flow cytometry was performed on Annexin V (fluorescein isothiocyanate labeled) and PI-stained MIO-M1 cells, challenged with *S. aureus* for the indicated time points. The bar graph represents the mean percentage of Annexin V and PI-positive cells (**c**). The *in vitro* data is a cumulative of three independent experiments performed in duplicates. **P<0.005; ***P<0.0005, *t*-test; GCL, ganglion cell layer; INL, inner nuclear layer; ONL, outer nuclear layer.

Increased retinal cell death has been associated with declined visual function in bacterial endophthalmitis.^14^ To determine whether *S. aureus* induces retinal cell death, *S. aureus*-infected B6 mouse retina was subjected to apoptosis assays. As shown in Figure 1a, *S. aureus* challenge significantly increased the number of TUNEL-positive cells, an indicator of apoptotic cell death. Moreover, the TUNEL-positive cells were observed in all retinal layers, including ganglion cell layer, inner nuclear layer and outer nuclear layer. In addition to bipolar cells, the predominant nuclei in inner nuclear layer belong to Müller glia, the major supporting cells spanning through the entire retina. To investigate whether Müller glia also died in response to bacterial infection, TUNEL and Annexin V and propidium iodide (PI) staining was performed on *S. aureus*-challenged human retinal Müller glia. To this end our results showed a time-dependent increase in Müller glia apoptosis (Figures 1b and c). Together, these findings suggest the induction of retinal cell apoptosis in bacterial endophthalmitis.

### *S. aureus* infection causes reduced mitochondrial membrane potential in retinal Müller glia

**Figure 2:**
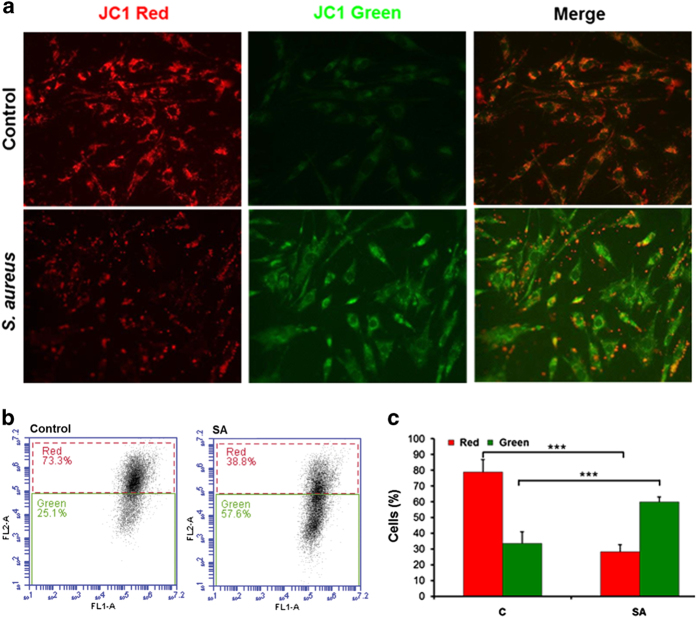
*S. aureus* challenge reduces the mitochondrial membrane potential in retinal Müller glia. MIO-M1 cells were left uninfected (control) or challenged with *S. aureus* (SA) RN6390 (multiplicity of infection, 10 : 1) for 8 h. Cells were stained for JC-1 to measure the change in mitochondrial membrane potential and observed under the fluorescence microscope (**a**). For quantification, flow cytometry was used to measure the relative fluorescence of free JC-1 (green) or aggregates JC-1 (red) in control *versus S. aureus-*challenged Müller glia (**b**). Note: the reduction in mitochondrial membrane potential corresponds to increase in green fluorescence and decrease in red fluorescence as shown in bar graphs (**c**). The results are representative of three independent experiments performed in duplicates. ***P<0.0005, *t*-test.

Recently, we discovered that Müller cells produce ROS as a part of their defense mechanism against *S. aureus* infection.^31^ Although ROS has an important role in innate defense, excessive ROS production may lead to cell death. This led us to investigate the role of mitochondria in *S. aureus*-induced retinal cell death including Müller glia. To assess the mitochondrial function following bacterial infection, we determined the mitochondrial membrane potential in Müller glia using JC-1 staining. As shown in Figure 2a, a red fluorescence was predominant in control cells, indicating the presence of JC-1 in the aggregated form in mitochondrial membranes. However, *S. aureus*-infected cells exhibited increased green fluorescence, indicating the existence of free JC-1 and the depolarized mitochondrial membrane potential. The JC-1 staining data was further confirmed by quantitative analysis of mitochondrial membrane depolarization using flow cytometry, showing decreased red fluorescence (JC-1 aggregates) and increased green fluorescence (free JC-1) in *S. aureus*-infected Müller glia as compared with uninfected control cells (Figures 2b and c).

### Cytochrome *c* release and Bax translocation are triggered in *S. aureus*-challenged retinal Müller glia

**Figure 3:**
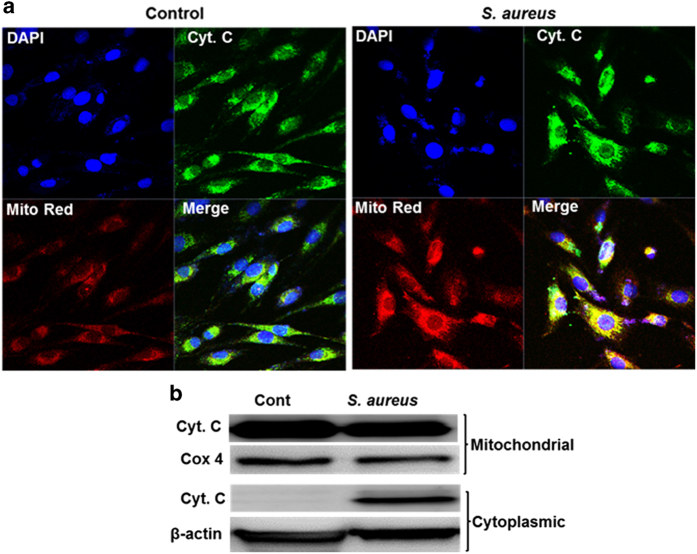
*S. aureus*-challenged retinal Müller glia releases cytochrome *c* from mitochondria. MIO-M1 cells were left uninfected (control) or challenged with *S. aureus* (SA) RN6390 (multiplicity of infection, 10 : 1) for 8 h. Cells were stained with MitoTracker red (Mito Red) dye followed by immunostaining for cytochrome *c* and observed under confocal microscope (**a**). In another experiment, *S. aureus*-challenged MIO-M1 cells were subjected to subcellular fractionation followed by western blot analysis of cytochrome *c* (**b**). Cox 4 and *β*-actin antibodies were used as protein loading controls for mitochondrial and cytoplasmic fractions, respectively. Results are representative of two independent experiments.

Following mitochondrial membrane depolarization, the release of cytochrome *c* from the mitochondria, a fundamental event in apoptosis, initiates the assembly of the apoptosome resulting in activation of initiator caspase-9, the downstream effector caspases-3 and ultimately cell death.^19,32^ We, therefore, investigated the cellular distribution of cytochrome *c* using confocal imaging and subcellular fractionation studies. To this end, our data show the presence of cytochrome *c* in the cytoplasm in *S. aureus*-challenged Müller glia as observed by confocal imaging (Figure 3a). Furthermore, the subcellular fractionation and western blot analysis revealed increased cytochrome *c* levels in cytosolic fractions of infected cells as compared with control cells (Figure 3b).

**Figure 4:**
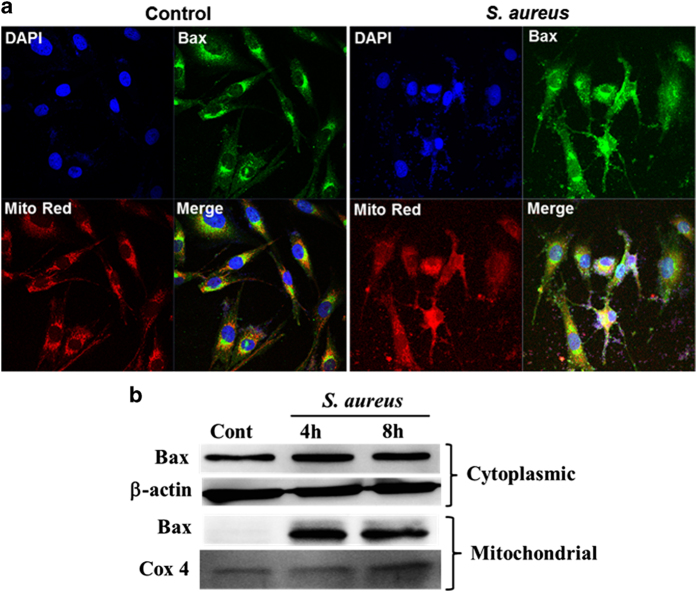
*S. aureus* induces mitochondrial translocation of Bax in retinal Müller glia. MIO-M1 cells were left uninfected (control) or challenged with *S. aureus* RN6390 (multiplicity of infection, 10 : 1) for the indicated time points. Cells were stained with MitoTracker red (Mito Red) followed by immunostaining for Bax and observed under confocal microscope (**a**). In another experiment, *S. aureus*-challenged MIO-M1 cells were subjected to subcellular fractionation followed by western blot analysis for Bax (**b**). Cox 4 and *β*-actin antibodies were used as protein loading controls for mitochondrial and cytoplasmic fractions, respectively. Results are representative of two independent experiments.

Having seen the release of cytochrome *c* in the cytoplasm, we next assessed the distribution of Bax, one of the major determinants of the mitochondrial release of cytochrome *c* and other apoptotic molecules such as Smac and AIF. Bax is generally sequestered in the cytosol and translocate into the mitochondria resulting into permeabilization of mitochondrial membrane and triggering the release of cytochrome *c* and the induction of apoptosis. As shown in Figure 4, challenge of Müller glia with *S. aureus* resulted into cellular redistribution of Bax with increased translocation of Bax from cytosol to the mitochondria (Figure 4a). These findings were further confirmed by subcellular fractionation and western blot analysis of Bax, showing increased presence of Bax in the mitochondrial fractions of both 4 and 8 h post *S. aureus*-infected cells (Figure 4b). Collectively, our data shows the redistribution of cytochrome c and Bax in response to *S. aureus* challenge.

### *S. aureus* infection initiates the activation of caspase-9 and -3 and cleavage of PARP-1 in the mouse retina and retinal Müller glia

**Figure 5:**
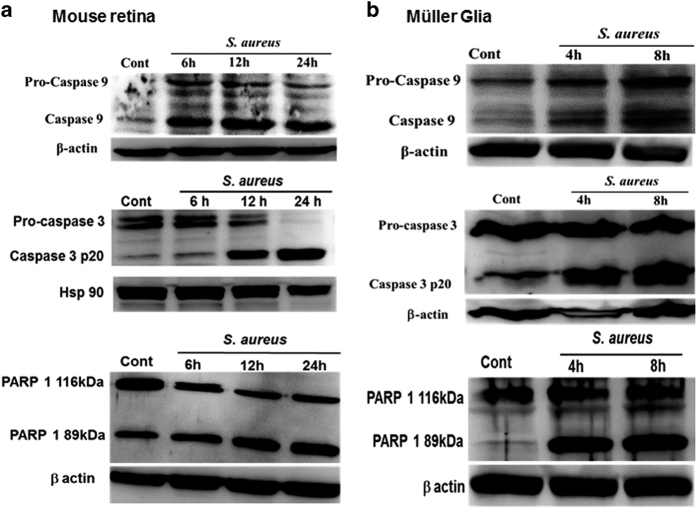
*S. aureus* infection initiates the activation of caspase-9 and -3 and the cleavage of PARP-1 in the mouse retina and retinal Müller glia. The retinal lysates, prepared from *S. aureus*-infected B6 mouse eyes at the indicated time point post infection were subjected to western blot analysis using caspase-9, caspase-3 and PARP-1-specific antibodies. The retinal tissue from PBS-injected eyes at 24 h was used as control (**a**). For *in vitro* studies, MIO-M1 cells were left uninfected (control) or challenged with *S. aureus* RN6390 (multiplicity of infection, 10 : 1) for indicated time periods. Cell lysates were prepared using radioimmunoprecipitation assay buffer containing protease and phosphatase inhibitors cocktail was used for the detection of caspase-9 and caspase-3 by western blot (**b**). Results are representative of two independent experiments.

After the release of cytochrome *c*, the next steps involve the proteolytic cleavage of pro-caspase-9 and -3 resulting into cell death. Similarly, the cleavage of PARP-1 by caspases is also considered to be a hallmark of apoptosis. We, therefore, investigated their potential involvement in triggering apoptosis in bacterial endophthalmitis. Indeed, our data shows that *S. aureus* induces proteolytic cleavage and activation of both pro-caspase-9 and -3 in the B6 mouse retina and the cultured retinal Müller glia (Figure 5). The time-course studies revealed that the activation of caspase-9 in retinal tissue is initiated as early as 6 h, further increase at 12 h, and slight decline at 24 h post *S. aureus* infection. However, the activation of caspase-3 that is, cleaved p20 levels proceeds at 12 h and being highest at 24 h (Figure 5a). Similarly, time-dependent activation of caspase-9 and -3 was observed in *S. aureus*-challenged Müller glia (Figure 5b). We also tested the activation of caspase-8 in both *in vivo* and *in vitro* experimental models, but we did not observe changes in caspase-8 (data not shown). PARP-1, a multifunctional nuclear protein, is one of the known cellular substrates of caspases. Therefore, we tested the PARP-1 cleavage following caspase activation. To this end, our data showed time-dependent cleavage of PARP-1 in *S. aureus*-infected retinal tissue, as evident by increased levels of the cleaved fragment (89 kDa) and simultaneous decrease of full size (116 kDa) PARP-1 (Figure 5a; lower panel). Similar, time-dependent cleavage of PARP-1 was detected in *S. aureus*-challenged retinal Müller glia (Figure 5b; lower panel). Together, these findings imply the involvement of caspase-9 and -3 activation and PARP-1 cleavage in executing the retinal cell death in endophthalmitis.

### *S. aureus* induces the release of AIF from mitochondria in retinal Müller glia

**Figure 6:**
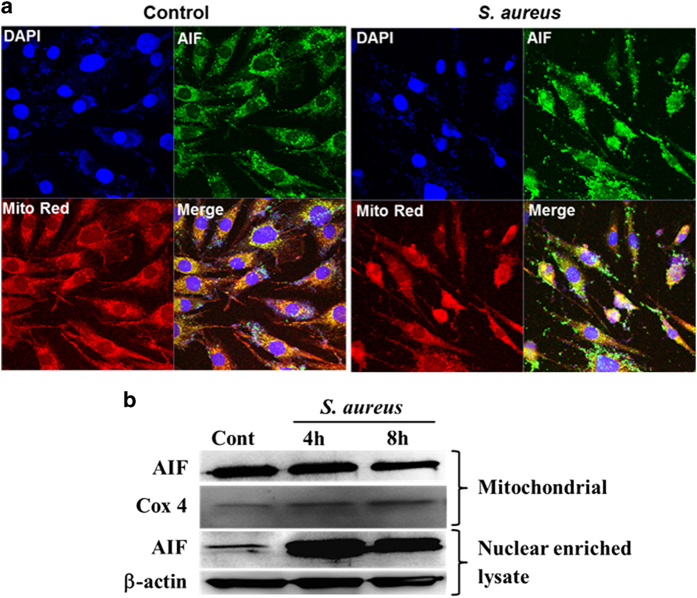
*S. aureus* induces release of AIF from mitochondria in retinal Müller glia. MIO-M1 cells were left uninfected (control) or challenged with *S. aureus* RN6390 (multiplicity of infection, 10 : 1) for the indicated time points. Cells were stained with MitoTracker red (Mito Red) followed by immunostaining for AIF and observed under confocal microscope (**a**). In another experiment, *S. aureus*-challenged MIO-M1 cells were subjected to subcellular fractionation followed by western blot for AIF (**b**). Cox 4 and *β*-actin antibodies were used as protein loading controls for mitochondrial and nuclear enriched fractions, respectively. Results are representative of two independent experiments.

PARP-1 activation followed by the release of AIF from mitochondria is considered to be an important step for caspase-independent apoptotic cell death.^33,34^ We investigated the cellular localization of AIF in retinal Müller glia. Our data show that *S. aureus* challenge induces the nuclear localization of AIF, as revealed by immunostaining and confocal imaging (Figure 6a) as well as by western blot analysis of AIF in subcellular fractions (Figure 6b). These results suggest the existence of caspase-independent apoptotic mechanism in endophthalmitis, involving the activation of PARP-1 activation followed by nuclear localization of AIF.

### Inhibition of caspase and PARP-1 activation ameliorates *S. aureus*-induced retinal cell death

**Figure 7:**
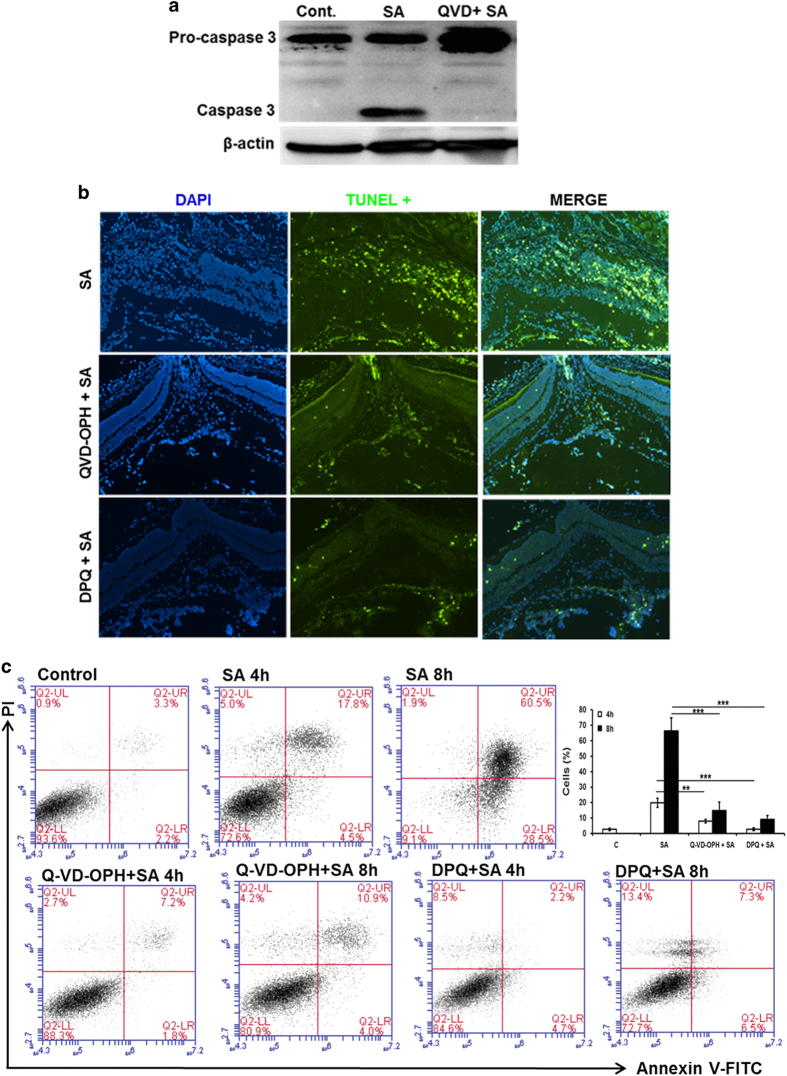
Inhibition of caspases and PARP-1 attenuates *S. aureus*-induced retinal cell apoptosis. The eyes of B6 mice (*n*=5) were pretreated with intravitreal administration of pan-caspase (Q-VD-OPH) or PARP-1 (DPQ) inhibitors (100 ng per eye for each) 12 h before the induction of staphylococcal endophthalmitis. Eye injected with DMSO (vehicle used to dissolve inhibitors) served as controls. After 24 h of *S. aureus* (SA) infection retina/eyes were subjected to western blot analysis for caspase-3 (**a**) or TUNEL staining to visualize apoptotic cells (**b**). The effect of caspase and PARP-1 inhibition on Müller glia apoptosis was assessed by flow cytometry of MIO-M1 cells pretreated with Q-VD-OPH (40 *μ*M) and DPQ (20 *μ*M), 1 h before *S. aureus* challenge for indicated time points (**c**). Results are representative of at least three independent experiments.**P<0.005; ***P<0.0005, *t*-test.

As both caspase and PARP-1 activation were identified as major contributors of apoptosis in *S. aureus* endophthalmitis, we sought to determine whether their inhibition can diminish retinal cell death. Following *in vitro* standardization of various inhibitors doses, we used a relatively non-toxic, broad-spectrum caspase inhibitor Q-VD-OPH^35–37^ and a PARP-1 inhibitor DPQ.^38–40^ First, we tested whether Q-VD-OPH treatment attenuates the activation of caspase-3 *in vivo*. As shown in Figure 7a, pre-treatment of mouse eyes with Q-VD-OPH before *S. aureus* infection almost completely inhibited the cleavage of caspase-3. Concomitantly, the number of TUNEL-positive cells were drastically reduced in the eye pretreated with Q-VD-OPH or DPQ (Figure 7b), suggesting the *in vivo* functionality of these inhibitors. Similar findings were observed *in vitro*, where both caspase and PARP-1 inhibitors significantly attenuated the apoptosis of retinal Müller glia in response to *S. aureus* challenge witnessed by quantitative flow cytometry analysis by Annexin V and PI staining (Figure 7c). Collectively, these results demonstrate caspases and PARP-1 as mediators of retinal cell death in response to microbial infection.

## Discussion

Bacterial endophthalmitis continues to be a major complication of ocular surgeries and remains an important cause of visual morbidity.^28,41^ Bacteria frequently found in endophthalmitis are *Staphylococcus epidermidis* or *S. aureus*.^42^ Clinical studies have shown that patients with *S. aureus* endophthalmitis are most likely to have severe vision loss, whereas endophthalmitis due to coagulase-negative staphylococci are generally milder and have a better outcome.^43^ In the past 5 years, studies from our laboratory have extensively investigated host–pathogen interactions in staphylococcal endophthalmitis.^13,14,26,44,45^ The increased severity of *S. aureus* endophthalmitis has been attributed to the ability of *S. aureus* to produce a wide range of virulence factors including extracellular and cell wall-associated proteins, which interact in multiple ways with retinal cells.^46^ More recently, using transcriptome and systems biology analyses, we established a molecular signature of *S. aureus* endophthalmitis.^47^ Among various pathways identified in the transcriptome study, the genes associated with the response to DNA damage, cell death and apoptosis were significantly upregulated in *S. aureus-*infected retina.^47^ These results also support our previous studies showing retinal cell death in bacterial endophthalmitis.^13,14^ Although, the induction apoptosis in microbial infection is not a new phenomenon, surprisingly, to our knowledge, none of the studies have delineated the molecular mechanisms of retinal cell death in endophthalmitis.

To explore the mode of *S. aureus*-induced retinal cell death, we started investigating the role of two well-known apoptotic pathways, the extrinsic/death receptor pathway and intrinsic/mitochondrial. The extrinsic signaling involves transmembrane receptor-mediated interaction and among them FasL/FasR and TNF-*α*/TNFR1 are the best characterized.^1^ FasL is constitutively expressed in the normal eye and has been shown to have an essential role in maintaining the immune-privileged environment by inducing the apoptosis in infiltrating inflammatory cells and evoking protection against ocular viral infections.^48^ In a recent study by Sugi *et al.*^49^ demonstrates that FasL is essential in promoting the resolution of inflammation and exerts protective effects in bacterial endophthalmitis. Although Pharmakakis *et al.*^50^ reported increased expression of FasL and Bax in a rat model of *S. epidermidis* endophthalmitis; their observation is limited to immunohistochemistry detection. Our qPCR analysis also showed upregulation of FasL and TNF-*α* in *S. aureus*-infected retina; however, we did not observe the activation of caspase-8 (data not shown), a major initiator caspase of extrinsic pathways. This suggests that extrinsic/death receptor pathway may not be a significant contributor of retinal cell death in *S. aureus* endophthalmitis.

As the retina is considered to be the most metabolically active (highly enriched in mitochondria) tissue in the body and our transcriptome analysis showing increased DNA damage response, we propose that apoptosis in bacterial endophthalmitis is primarily via an intrinsic pathway. Moreover, we recently discovered that retinal Müller glia generates ROS in response to *S. aureus* challenge^29^ and ROS are potent inducers of oxidative damage and have been implicated in regulation of apoptosis including the retina.^51^ The mitochondria being the major source of ROS production further propelled our interest to assess their contribution in retinal cell death in bacterial endophthalmitis. Mitochondrial membrane depolarization followed by the release of pro-apoptotic factors is the major consequence of the mitochondria-mediated apoptosis. Indeed, our data show the depolarization of mitochondrial membrane (reduced JC-1 aggregates) in bacterial-challenged Müller glia and the concomitant release of cytochrome *c* in the cytoplasm. Similarly, the subcellular fractionation analysis of *S. aureus*-infected retinal tissue showed the presence of cytochrome *c* in the cytoplasmic fraction. Another mechanism leading to alterations of the mitochondrial membrane integrity is the translocation of pro-apoptotic protein Bax from the cytosol into mitochondria where they form channels and/or regulate the function of pre-existing channels.^1^ It has been reported that the translocation of Bax from cytosol into mitochondria targets the mitochondrial membrane contact sites, causing the mitochondrial permeability transition, loss of mitochondrial potential, release of cytochrome *c*, subsequent activation of caspases and DNA fragmentation, resulting into apoptotic cell death.^18,52,53^ We, therefore, investigated the subcellular redistribution of pro-apoptotic Bax and our results clearly demonstrate the translocation of Bax from cytosol into the mitochondria following *S. aureus* infection.

The downstream apoptotic events in intrinsic pathway following the release of cytochrome *c* and Bax translocation involve caspase activation. Indeed, our data showed the activation of caspase-9 and caspase-3 in *S. aureus*-infected Müller glia and the mouse retina. A significant increase in the cleavage of caspase-3 along with DNA fragmentation is consistently observed among various retinal diseases, including cytomegalovirus retinitis.^54,55^ An earlier study by Whiston *et al.*^56^ also showed the involvement of caspase-3 in *S. aureus*-induced apoptosis and its inhibition by *αβ* crystalline protein. A number of different apoptotic pathways may lead to activation of caspase-3-mediated apoptosis therefore, a mechanistic evaluation of retinal cell apoptosis is mandatory to evaluate the therapeutic intervention of cell death to protect retina and vision loss in bacterial endophthalmitis. In this study using the complementary *in vitro* and *in vivo* models of *S. aureus* infection, our data support the involvement of mitochondria-mediated caspase-3 activation in inducing retinal cell death in bacterial endophthalmitis.

PARP-1 is a nuclear enzyme also called as guardian of the genome acting as a sentinel for genome damage.^57^ However, PARP-1 has been reported to have an important role in cell death in various disease models in both caspase-dependent and -independent manners.^57–62^ PARP-1 selectively activated by DNA strand breaks and proteolytic cleavage of PARP-1 has been considered as a hallmark biochemical feature of apoptosis. Our data show that *S. aureus* induces the cleavage of PARP-1 in retina and retinal cells indicating the involvement of PARP-1 in retinal apoptosis. Although, the cleavage of PARP-1 indicate the caspase-dependent apoptosis, its activation coupled with the translocation of AIF to the nucleus may suggest caspase-independent cell death.^58,63^ Our data showing the subcellular redistribution (translocation from mitochondria to nucleus) of AIF by immunostaining and subcellular fractionation indicate the role of caspase-independent mechanisms of retinal cell death in endophthalmitis.

As the caspase and PARP-1 activation were identified as key regulators of apoptosis in *S. aureus* endophthalmitis, therefore, we tested whether pharmacological inhibition of caspase and PARP-1 activation can prevent retinal apoptosis. We tested a relatively non-toxic, broad-spectrum caspase inhibitor Q-VD-OPH^35–37^ and our data showed significant attenuation of apoptotic retinal cells in eyes pretreated with caspase inhibitors. Similarly PARP-1 inhibitor DPQ^38–40^ also showed a significant reduction in TUNEL and Annexin V and PI-positive cells in the mouse retina and cultured retinal Müller glia. These findings suggest that *S. aureus*-induced apoptosis in the retina, and retinal cells is mediated by the activation of caspases and PARP-1 and that their pharmacological inhibition can be harnessed as the suitable therapeutic target to prevent retinal cell apoptosis.

In summary, to the best of our knowledge, using both *in vivo* and *in vitro* models, we elucidate here for the first time, detailed mechanisms of *S. aureus*-induced retinal cell apoptosis in bacterial endophthalmitis. We report that *S. aureus*-induced mitochondrial membrane depolarization, causing the release of cytochrome *c*, and the translocation of Bax to the mitochondria. These signaling events culminated to the activation of caspase-9 and -3. The release of AIF and the cleavage of PARP-1 were also observed in *S. aureus*-infected retinal cells. Together, these findings led to the conclusion that retinal cell apoptosis in bacterial endophthalmitis is mediated by caspase-dependent and independent mitochondrial pathways.

## Materials and Methods

### Bacterial strain and reagents

*S. aureus* (strain RN6390) was maintained in tryptic soy broth (Sigma-Aldrich, St. Louis, MO, USA). Antibodies against caspases (caspase-3: Sc7148; caspase-9: Sc8355), cytochrome *c* (Sc7159), AIF (Sc5586), PARP-1 (Sc25780), Bax (Sc526) and Cox 4 (Sc292052) were purchased from Santa Cruz Biotechnology (Paso Robles, CA, USA). A mouse monoclonal anti-*β*-actin (A2228) antibody was purchased from Sigma-Aldrich. Secondary horseradish peroxidase-conjugated anti-mouse (170–6516) or anti-rabbit (170–6515) IgG antibodies were purchased from Bio-Rad (Hercules, CA, USA). Annexin V and PI staining kit, and JC-1 staining kits were purchased from BD Biosciences (San Jose, CA, USA). Caspase and PARP-1 inhibitors were purchased from R&D Biosciences (Minneapolis, MN, USA). ApopTag Fluorescein *In situ* Apoptosis Detection Kit was purchased from Millipore (Billerica, MA, USA).

### Cell culture and infection

The immortalized human Müller glia cell line MIO-M1 (received from Dr. Astrid Limb, University College, London, UK) was maintained in DMEM supplemented with 10% FBS, 1% penicillin–streptomycin and 10 *μ*g/ml l-glutamine. Whenever needed, cells were grown overnight in low serum (1–2%) and antibiotic-free DMEM before infection. The cells were challenged with *S. aureus* RN6390 with multiplicity of infection 10 : 1 for entire study. The cells were treated with caspase (Q-VD-OPH, 40 *μ*M) and PARP-1 (DPQ, 20 *μ*M) inhibitors 1 h before infection for inhibition studies.

### Mice and ethics statement

C57BL/6 (B6) mice were purchased from Jackson’s laboratory (Bar Harbor, ME, USA). Animals were housed in a restricted access DLAR facility at the Kresge Eye Institute, were maintained in a 12 h light:12 h dark cycle, and fed on LabDiet rodent chow (Labdiet Pico lab Laboratory, St Louis, MO, USA) and water *ad libitum*. Mice were treated in compliance with the Association for Research in Vision and Ophthalmology (ARVO) Statement for the Use of Animals in Ophthalmic and Vision Research, and all procedures were approved by the Institutional Animal Care and Use Committee (IACUC) of Wayne State University under protocol # A 08-02-13.

### Induction of endophthalmitis

Endophthalmitis was induced in B6 mice by intravitreal inoculation of 5000 c.f.u. of *S. aureus* RN6390 as described earlier.^13,64^ The vehicle (PBS/DMSO)-treated eyes served as controls. The caspase and PARP-1 inhibitors (100 ng per eye) were injected intravitreally, 12 h before bacterial inoculation for the inhibition studies. At the desired time points post infection; retinas from the enucleated eyes were subjected to western blotting (retinal lysates made in PBS-containing protease and phosphatase inhibitor cocktail) or TUNEL staining as described in following sections.

### TUNEL

Apoptosis was assessed by TUNEL staining. For *in vitro* studies, MIO-M1 cells were grown and challenged with *S. aureus* in a four-well chamber slide (Fisher Scientific, Rochester, NY, USA) for indicated time points. For *in vivo* studies, following infection, the eyes were fixed in Tissue-Tek OCT (Sakura, Torrance, CA, USA) and 5 *µ*m-thick sagittal sections were collected from each eye and mounted onto microscope slides. TUNEL staining was performed on MIO-M1 cells as well as retinal cryosections using ApopTag Fluorescein In situ Apoptosis Detection Kit as per the manufacturer’s instruction (Millipore). The TUNEL stained cells/retinal sections were visualized using an Eclipse 90i fluorescence microscope (Nikon, Melville, NY, USA).

### Annexin V and PI staining

Annexin V and PI staining was carried out using a commercial apoptosis assay kit (BD Biosciences, San Jose, CA, USA) as per manufacturer’s recommendations. Briefly, cells were grown and challenged with *S. aureus* in a six-well plate for indicated time points. Following challenge, cells were washed with PBS and harvested by treating with TrypLE (Thermo Scientific, Rockford, IL, USA). The cells were washed with PBS followed by washing with 1×-Annexin V binding buffer and incubated for 30 min in the dark in 100 *μ*l Annexin V binding buffer containing 5 *μ*l fluorescein isothiocyanate-labeled Annexin V and 5 *μ*l PI. Following incubation cells were washed with 1×-Annexin V binding buffer and acquired by a BD AccuriC6 flow cytometer (BD Biosciences, Ann Arbor, MI). At least 50 000 cells were analyzed in each treatment. The data were analyzed using AccuriC6 software (BD Biosciences, Ann Arbor, MI).

### JC-1 staining

Changes in mitochondrial membrane potential were assessed by JC-1 staining as per manufacturer’s instruction (BD Biosciences). In brief, cells were cultured in a four-chamber slide and challenged with *S. aureus* for the indicated time points. Following challenge, cells were washed with PBS and incubated with BD MitoScreen (JC-1) (BD Biosciences) for 1 h. Cells were then visualized using an Eclipse 90i fluorescence microscope (Nikon). For quantitative analysis, cells were grown in a six-well plate and challenged with *S. aureus* RN6390 for indicated time periods. Cells were harvested by treating with TrypLE, washed and stained with JC-1 dye as indicated above. Cells were acquired by AccuriC6 flow cytometer with excitation at 488 nm and emission using 670 nm. At least 50 000 cells were analyzed in each treatment. The flow cytometric data were analyzed using AccuriC6 software.

### Subcellular fractionation

Subcellular fractionations were performed in order to study the localization of cytochrome *c*, Bax and AIF. Nuclear, cytosolic and mitochondrial fractions were prepared as follows: following challenge the cells were re-suspended and scraped in 500 *μ*l of subcellular fractionation buffer (20 mM HEPES (pH 7.4), 250 mM sucrose, 10 mM KCl, 1.5 mM MgCl_2_, 1 mM EDTA, 1 mM EGTA, 1 mM DTT, and protease and phosphatase inhibitor cocktail). The cell lysates were passed through a 25 G needle, 5–10 times using 1 ml syringe and incubated on ice for 30 min. The lysates were centrifuged at 720×*g* for 5 min at 4 °C to separate nuclear fraction (pellet) and washed three times with ice-cold PBS. The resulting supernatants containing mitochondrial and cytosolic fraction were centrifuged again at 10 000×*g* for 15 min at 4 °C. The resulting supernatants were used as the cytosolic fraction. The pellet was washed three times in ice-cold PBS and used for mitochondrial fraction.

### Western blotting

Following *S. aureus* challenge, MIO-M1 cells were lysed with radioimmunoprecipitation assay buffer (50 mM Tris-HCl (pH 8.0), 150 mM NaCl, 1.0% NP-40, 0.5% sodium deoxycholate, 0.1% sodium dodecyl sulfate (SDS), 100 mM sodium pyrophosphate, and 3.5 mM sodium orthovanadate). A protease inhibitor cocktail containing aprotinin, pepstatin A, leupeptin and antipain (1 mg/ml each), and 0.1 M phenylmethylsulfonyl fluoride (Sigma-Aldrich) were added to the radioimmunoprecipitation assay buffer before use (1 *μ*l/ml). Retinal lysates were also prepared in PBS-containing protease and phosphatase inhibitor cocktail by sonication followed by centrifugation at 12 000×*g* for 15 min. The total protein concentration of the cell and retinal lysates were determined using a Micro BCA protein assay kit (Thermo Scientific, Rockford, IL, USA). Total protein samples (30–50 *μ*g) were resolved on SDS-PAGE in Tris-glycine-SDS buffer (25 mM Tris, 250 mM glycine and 0.1% SDS) and electro-blotted onto a polyvinylidene fluoride membrane (Millipore). After blocking for 1 h in 5% MPBST (PBS-containing 0.05% Tween 20 and 5% nonfat milk), the blots were probed with primary antibodies (1 : 1000) overnight at 4 °C. The membranes were washed three times with PBST (PBS-containing 0.05% Tween 20) and incubated with horseradish peroxidase-conjugated secondary antibodies (Bio-Rad, Hercules, CA, USA) diluted in 5% MPBST at RT for 1 h. Protein bands were visualized with Supersignal West Femto Chemiluminescent Substrate (Thermo Scientific).

### Fluorescence staining and confocal imaging

MIO-M1 cells were cultured in a four-well chamber slide (Fisher Scientific, Rochester, NY, USA) and stimulated with *S. aureus* for 8 h. Following challenge cells were stained with MitoTracker red as per manufacturer instructions (Thermo Scientific). Following staining, the cells were washed three times with PBS and fixed with 4% paraformaldehyde made in PBS for 15 min. After washing, the cells were permeabilized with 0.2% Triton X-100 made in PBS. The fixed and permeabilized cells were then blocked with 1% (w/v) BSA containing 0.2% Triton X-100 for 1 h at room temperature, followed by incubation with primary antibodies (1 : 100 dilution) overnight at 4 °C. Following removal of the primary antibodies, the cells were washed extensively with PBS and incubated for 1 h with specific fluorescein isothiocyanate-conjugated secondary antibodies (1 : 200 dilutions) at room temperature. Finally, the cells were extensively washed with PBS, and the slides were mounted in Vectashield anti-fade mounting medium (Vector Laboratories, Burlingame, CA, USA) and visualized using confocal laser scanning microscope (Leica TCS SP 8; Leica Microsystems, Buffalo Grove, IL, USA).

### Statistical analysis

All data are expressed as the mean±S.D. unless indicated otherwise. Statistical differences between experimental groups were determined using Student’s *t*-test. All statistical analyses were performed using GraphPad Prism 6.2 (GraphPad Software, La Jolla, CA, USA). A value of *P*<0.05 was considered statistically significant.
